# White Rabbit in radio interferometry

**DOI:** 10.1007/s10686-025-10038-4

**Published:** 2026-01-03

**Authors:** E. Paul Boven, Jeroen C. J. Koelemeij, Chantal van Tour, Rob Smets, Rodrigo González Escudero, Huib Jan van Langevelde

**Affiliations:** 1 JIVE - Joint Institute for VLBI ERIC, Oude Hoogeveensedijk 4, Dwingeloo, 7991PD the Netherlands; 2 Leiden Observatory, PO box 9513, Leiden, 2300RA the Netherlands; 3 Vrije Universiteit Amsterdam, De Boelelaan 1100, 1081HZ Amsterdam, the Netherlands; 4 OPNT bv, Delftechpark 1, Delft, 2628XJ the Netherlands; 5 SURF, PO Box 19035, 3511EP Utrecht, the Netherlands; 6 CAMRAS, Oude Hoogeveensedijk 4, 7991PD Dwingeloo, the Netherlands; 7 TNO, Anna van Buerenplein 1, 2595DA Den Haag, the Netherlands

**Keywords:** VLBI, Interferometry, Radio Astronomy, White Rabbit, Allan deviation

## Abstract

Radio interferometry relies on distributed telescopes having precise time and frequency sources that allow them to operate coherently over timescales up to several hours. As radio telescopes are being connected to fiber-based high-speed communication networks, it is of interest to make use of these for time and frequency distribution. The White Rabbit protocol enables the accurate and precise distribution of time and frequency signals over telecommunication optical fibers. We set out to evaluate the quantitative limits for interferometers over a range of observing frequencies when synchronized through White Rabbit. We develop a method to quantify the possible loss of sensitivity due to the phase noise contribution of a White Rabbit link. Our findings include a new expression for the coherence loss due to flicker phase noise. As this type of noise is common in frequency transfer links, its use extends beyond the case of White Rabbit. Furthermore, we designed a calibration procedure to measure the dispersion on already deployed fiber networks. We demonstrate adding a White Rabbit signal to an existing high-speed production network, together with data traffic on other wavelengths on the same fiber. Finally we built a VLBI setup with fiber links of 35 and 169 km, connecting two radio telescopes together. The agreement between our predicted and measured coherence loss indicates the usefulness of our approach, and that White Rabbit is suitable for clock distribution in radio interferometry instruments. We find that regular White Rabbit v3 switches support observing frequencies up to 3.5 GHz, and their low-jitter version up to 15 GHz.

## Introduction

Radio interferometry [24] is a technique where multiple radio telescopes are simultaneously observing the same area of the sky, capturing the same frequency range. By combining the received signals, images of the radio sky can be made with a resolution comparable to what could be achieved by an antenna spanning the largest distance between the receptors. In VLBI (very long baseline interferometry, [8]) the baselines between antennas can be hundreds to even thousands of kilometers in length. Whereas smaller scale radio interferometers can distribute a phase reference signal amongst their members, in VLBI the stations are traditionally equipped with their own atomic frequency standard with sufficient phase stability. For cm wavelength VLBI, these are usually hydrogen maser atomic clocks.

White Rabbit [32] is a system for the reliable distribution of time and frequency signals and data over standard telecommunications single mode glass-fiber. It has been developed at CERN (Conseil Européen pour la Recherche Nuclèaire, European council for nuclear research) for the synchronization of the LHC (Large Hadron Collider) particle accelerator, and published under their open hardware license. It is based on existing standardized technologies such as Ethernet, precision time protocol (PTP) and optical small form-factor pluggable (SFP) modules. The open source nature of the project has made it possible to extend the functionality and reach of White Rabbit, and there is a lively community of users in fields such as particle physics, finance and astronomy. As White Rabbit is an emerging standardized, off-the-shelf solution for the distribution of timing signals over fiber infrastructure, we set ourselves the goal of examining its application for the distribution of reference timing signals for radio interferometers. With the expected release of a 10 Gb/s and even 25 Gb/s capability for White Rabbit, this offers the tantalizing prospect of transporting both the timing to, and the digitized received radio spectrum from each of the elements of a radio interferometer, using only a single fiber connection per antenna.

The phase noise due to the White Rabbit link [20, 21] determines the performance of an interferometer employing such a frequency distribution link. Insufficient phase stability of the reference phase distributed to the antennas in a radio interferometer leads to a loss of sensitivity, due to a reduction in amplitude of the cross product calculated in the correlator. Observations generally use a coherent averaging time of seconds to minutes. Especially in VLBI, the geographically uncorrelated turbulence in the ionosphere and troposphere will destroy the phase coherence between antennas on timescales longer than a few minutes, so we are mostly interested in the behavior of the White Rabbit link up to this timescale. To remove these longer term phase fluctuations due to the atmosphere, the interferometer will regularly observe a bright, compact radio source to re-calibrate the phase differences.

Following [22], the coherence *C* as function of integration time *T* is defined as

1 $$\begin{aligned} C(T) = \left| \frac{1}{T}\int _{0}^{T} e^{j\phi (t)}\textrm{d}t \right| , \end{aligned}$$

where $\phi (t)$ describes the instrumental phase variations between two radio telescopes, at the observing frequency. *C*(*T*) ranges from 1 (for perfect coherence) down to 0 (for complete loss of coherence).

The relative phase variations $\phi (t)$ can be expressed as a power spectral density (PSD) $S_\phi (f)$. As radio telescopes can generally operate at multiple frequencies, it is useful to express the frequency spectrum of the phase reference noise variations in terms of the fractional frequency variations $S_y(f)$, where $S_y(f) = \frac{f^2}{\nu _0^2} S_\phi (f)$, with *f* the frequency offset from the carrier, and $\nu _0$ the observing frequency.

The different physical processes responsible for the phase noise will each result in a different slope (on a log-log plot) of the fractional frequency PSD $S_y(f) = \Sigma h_af^a$, with *a* an integer value (between -4 and 3, inclusive) characteristic for the noise process in question, and $h_a$ a measure of the amplitude of the fractional frequency noise for that process. This leads to a simple, statistical description of the noise processes.

When we can assume that the phase variations are due to a stationary Gaussian process, the coherence loss $L_C$ will be:

2 $$\begin{aligned} L_{C}(T) = 1 - \sqrt{\langle C^{2}(T)\rangle }, \end{aligned}$$

where the angle brackets indicate the mean. The goal of the reference phase distribution in an interferometer is to have the coherence loss negligible compared to the other limits on its sensitivity. For the SKA (Square Kilometre Array) phase 1 design, the coherence budget allows for a loss of up to 2% for integration times of 1 s and 60 s [1], which we adopt as a goal for this publication.

The coherence of a radio interferometer is affected by the long term reference phase stability. In the phase noise PSD, the slow phase variations that can destroy the coherence are not easily visible, and the Allan deviation (ADEV) [2] offers a more convenient way to express the long term stability of a signal. The Allan deviation expresses the 2-sample RMS of the phase difference between two clock signals, as a function of the integration time. The phase difference between two clocks is the quantity which determines the achievable coherence. The literature on determining the coherence loss from the Allan deviation of the phase reference signal is well established [26]. Coherence is only possible when the slope of the ADEV is negative, which is the case for white phase noise (WPN, $a=2$), flicker phase noise (FPN, $a=1$) and white frequency noise (WFN, $a=0$). For WPN and WFN the coherence loss as function of the ADEV is known, and we introduce an expression to calculate the loss due to FPN in the next section.

A White Rabbit link is a frequency transfer system, and as such a two-port system, [23]. The phase noise contribution of such systems is limited to WPN and FPN, as any longer term variations in phase can only be due to variations in delay through the system, which even in a WR link spanning more than 100 km are still strictly limited. The WR system itself is designed to counteract any delay changes on the fiber. This does not completely rule out slower phase drift due to e.g. diurnal temperature effects or aging of components, but these are at a much longer timescale and will have no impact on the coherence loss for the timescales that we are interested in.

In the next sections we will present a method to determine the coherence loss as a function of the ADEV noise contribution due to the WR link, the observing frequency, and the integration time of the interferometer. After that, we will examine how to extend the range of a WR link by making use of existing, in-use fibers, and show a method for calibrating the dispersion coefficient on already deployed fiber links. In the final section we illustrate our methods by performing actual VLBI observations, with the phase reference distributed over such a White Rabbit link.

**Fig. 1:**
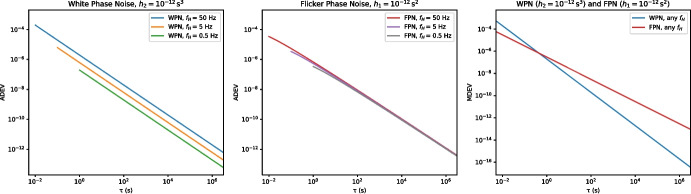
The effects of measurement bandwidth and noise process on ADEV and MDEV graphs, for noise signals wider than the measurement bandwidth

## Coherence and Allan deviation

The Allan variance $\sigma _y^2(\tau )$ (AVAR) or Allan deviation $\sigma _y(\tau )$ (ADEV) describes the statistical behavior of the phase difference between two signals such as e.g. atomic clocks, and can be readily measured with commercial off-the-shelf equipment. Our goal is to derive the expected coherence loss in an interferometric observation from such measurements on a White Rabbit link.

To calculate the ADEV, one typically uses a series of phase difference or frequency measurements which are separated by a fixed interval in time. This is a sampling process, and in order to prevent aliasing [9, 30], the bandwidth of the phase noise variations must first be filtered such that they fall within the first Nyquist zone. This bandwidth limit is known as the equivalent noise bandwidth (ENBW) of the measurement. A system that produces deviation measurements e.g. once every second, will have an ENBW of $0.5\,\textrm{Hz}$. Initially, we will assume that the bandwidth of the noise signal being measured is larger than the measurement bandwidth $f_h$.

Although the ADEV is the most common way to express the stability of atomic clocks and similar systems, it has two well known drawbacks in our application. First of all, both the white phase noise and the flicker phase noise processes result in a slope of $\approx -1$ on a log-log plot, rendering them indistinguishable in an ADEV plot. Secondly, both for the WPN and FPN cases, the measured result depends on the bandwidth $f_h$ used for the measurement. For ADEV measurements of WPN and FPN, it is therefore crucial to always state the measurement bandwidth.

The modified Allan deviation (MDEV) [3] $^M\sigma _y(\tau )$ does not suffer from these shortcomings, as it employs a frequency cut-off $f_h$ which scales with the integration time $\tau$. Figure 1 shows these effects on simulated WPN and FPN data.

Phase noise measurement equipment can usually display its results both as an ADEV and MDEV. As the MDEV is insensitive to $f_h$, this allows for a more direct determination of $h_a$ for each of the phase noise types present.

If the phase noise contribution of a frequency transfer link behaves like white phase noise, the Allan variance $\sigma ^2_{y}(\tau )$ [4] and modified Allan variance $^M\sigma ^2_y(\tau )$ [5] can be expressed as:

3 $$\begin{aligned} \sigma ^2_{y}(\tau ) = \frac{3 f_h}{4\pi ^2}\frac{h_2}{\tau ^2},\hspace{1em} ^M\sigma ^2_y(\tau ) = \frac{3}{8\pi ^2}\frac{h_2}{\tau ^3} \end{aligned}$$

with $\tau$ the integration time in seconds, $h_2$ the RMS deviation of the WPN, and $f_h$ the noise bandwidth of the measurement. The coherence loss as function of the observing frequency $\nu _0$ is then:

4 $$\begin{aligned} L_{C,\textrm{WPN}} = 1 - \sqrt{e^{-h_2f_h\nu _0^2}} \end{aligned}$$

For flicker phase noise, the Allan variance and modified Allan variance are

5 $$\begin{aligned} {\begin{matrix} \sigma ^2_y(\tau ) & = \frac{3\gamma - \ln 2 + 3 \ln (2\pi f_h\tau )}{4\pi ^2}\frac{h_1}{\tau ^2} \\ ^M\sigma ^2_y(\tau ) & = \frac{24 \ln 2 - 9 \ln 3}{8\pi ^2}\frac{h_1}{\tau ^2} \end{matrix}} \end{aligned}$$

The symbol $\gamma$ represents the Euler-Mascheroni constant, $0.57721\cdots$. Expressions to calculate the coherence loss for the cases of white phase noise (see above) and white frequency noise are in the literature [26]. In Appendix A we derive a closed-form expression for the coherence loss in the case of flicker phase noise:

6 $$\begin{aligned} L_{C,\textrm{FPN}} = 1 - \sqrt{\frac{2(2\pi e^\gamma f_hT)^{-h_1\nu _0^2}}{(1 - h_1\nu _0^2)(2-h_1\nu _0^2)}} \end{aligned}$$

The coherence loss is again sensitive to the noise bandwidth $f_h$, although much less so than in the white phase noise case as the phase noise spectrum for flicker phase noise has a negative slope, limiting the contribution of the higher modulation frequencies. For flicker phase noise, the coherence loss also depends on the integration time *T* of the interferometer; longer integrations will lose some sensitivity.

**Fig. 2:**
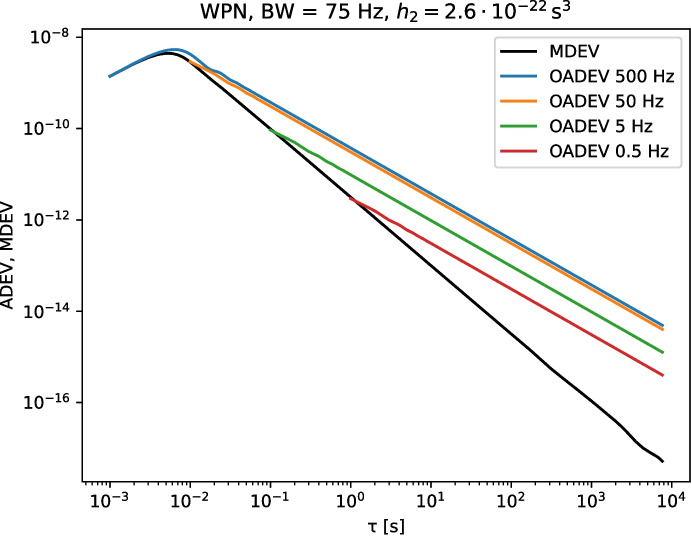
The ADEV and MDEV for a simulated bandwidth limited WPN signal. The oadev traces show the overlapping ADEV for the given ENBW

White phase noise is characterized by a constant amplitude of the phase noise as function of frequency when plotting $S_\varphi (f)$, showing phase noise against frequency. A flat phase noise spectrum into infinity however is not physically possible, and in practice it is always limited to some highest frequency. When using a high ENBW to measure a phase noise signal which itself has a low bandwidth, the resulting ADEV will have a turnover at the shortest integration times (which are only reachable with a high ENBW). The measured ADEV will no longer increase with the measurement bandwidth once the complete phase noise signal is captured with a sufficiently high ENBW. Both effects are visible in Fig. 2. To calculate the coherence loss in the case of WPN, one should use the actual signal bandwidth when it is lower than the measurement ENBW [30]. The effect of the noise bandwidth on FPN signals is much smaller, as can be seen in Fig. 1.

In the next section, we will see that the phase noise contribution of White Rabbit switches can be described as a combination of a bandwidth limited white phase noise, and a flicker phase noise contribution.

### ADEV measurements

The White Rabbit switch WRS-3/18 is available in two variants: The original design [25], and a version with improved phase noise performance which is equipped with a ‘low jitter daughter-board’ (LJD) [21]. We have measured the ADEV performance of both using a Microsemi 3120a phase noise analyzer. In both cases, the 10 MHz and 1 PPS inputs to the WR switch, and the reference input to the 3120a, were sourced from a SRS FS725 rubidium atomic clock. Any phase noise due to the FS725 is not a factor because the 3120a measures the difference in phase between its inputs (i.e. the input and the output of the WR link 10 MHz signal), and the round-trip time through the WR switches and short fiber is too short to expect de-coherence. The WR switches were connected using 1000base-BX10 BiDi optics, and a short (2 m) fiber. All measurements were done at the 10 MHz (CLK2) output of the WR switch at the end of the fiber link.

**Fig. 3:**
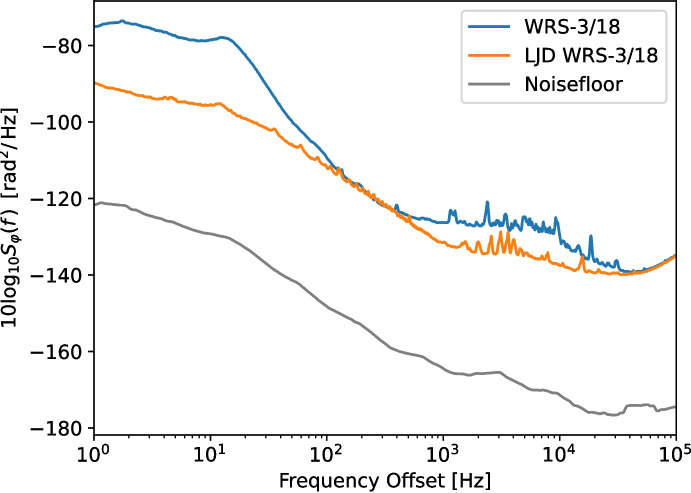
Phase noise measurement for both switch types. The noise floor is a smoothed average of the FFT of the imaginary component of the measurement traces, re-implementing the instrument reported noise floor

**Fig. 4:**
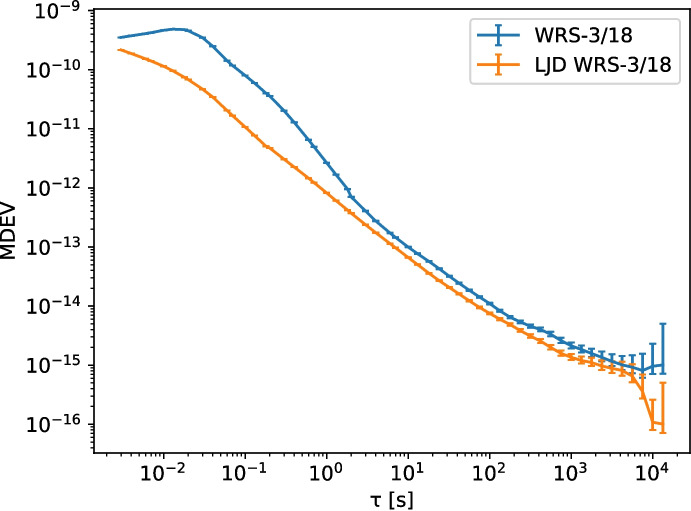
MDEV for both switch types. Error-bars are $1\sigma$ as calculated using edf_greenhall() [11] and confidence_interval() in Allantools

As shown in Fig. 3, the phase noise of the original White Rabbit switch (the WRS-3/18) is higher than in its low-jitter counterpart, especially for modulation frequencies below 20 Hz. The slope for the non-LJD link in the MDEV plot in Fig. 4 around $\tau = 1\,\textrm{s}$ and lower is -1.5, indicating that the dominant noise process there is white phase noise.

The introduction of the Low Jitter Daughter-board extension to the switches cleans up most of the white phase noise below 20 Hz found in the ‘regular’ WRS. The MDEV plot shows that the phase noise in this case has a slope of -1 over most of the range, indicating that it is dominated by flicker phase noise, most likely from its main timing oscillator.

For both types of WR switch, we now characterize the link phase noise contribution as a combination of WPN and FPN. First, in the MDEV plots, we select the areas with a slope of $-1$ (FPN) and $-1\frac{1}{2}$ (WPN). By fitting a line with the appropriate slope over the MDEV measurements (in log-log space), we determine $h_2$ and $h_1$. Then, from the ADEV plot with the highest ENBW, we determine the bandwidth of the noise contribution, which we designate as $\textrm{bw2}$ for the WPN. Note that this method is only allowed when $\text {ENBW} \gg f_h$ [30].

The effect of $f_h$ in the case of FPN is very small, and is obscured by the WPN contribution to the ADEV and MDEV. Given the limited effect of $f_h$ on FPN signals, we will assume it to be equal to the ENBW of the measurement. The results of these fits are summarized in Table 1.

**Table 1:** Phase noise process parameters from the fit, including statistical uncertainties

	WPN		FPN
	$h_2$	bw2	$h_1$
Regular	$1.869(25) \cdot 10^{-22}\,\mathrm {s^3}$	17.42(5) Hz	$1.479(16) \cdot 10^{-23}\,\mathrm {s^2}$
LJD	$3.48(3) \cdot 10^{-24}\,\mathrm{s^3}$	25.9(1) Hz	$7.14(12) \cdot 10^{-24}\,\mathrm {s^2}$

### Simulation of the phase noise and coherence loss

To judge the quality of our phase noise model, we use the Python module Allantools [31] to create a simulated phase noise signal with the appropriate FPN and WPN contributions. To be compatible with our recorded ADEV measurements, we choose the same set of ENBW values (500, 50, 5 and 0.5 Hz) for this simulation.

The phase noise generator implemented in the Python package Allantools is based on the procedure described by [15]. The input parameters to the phase noise generator are the slope of the phase noise PSD *b*, and the discrete variance $Q_d$. The value of *b* is related to *a* (the slope of the fractional frequency PSD) as $a = b + 2$. The discrete variance $Q_d$ can be calculated from $h_a$ and the measurement bandwidth $f_h$:

7 $$\begin{aligned} Q_d = \frac{h_a}{4} \pi ^{-a} f_h^{a-1} \end{aligned}$$

**Fig. 5:**
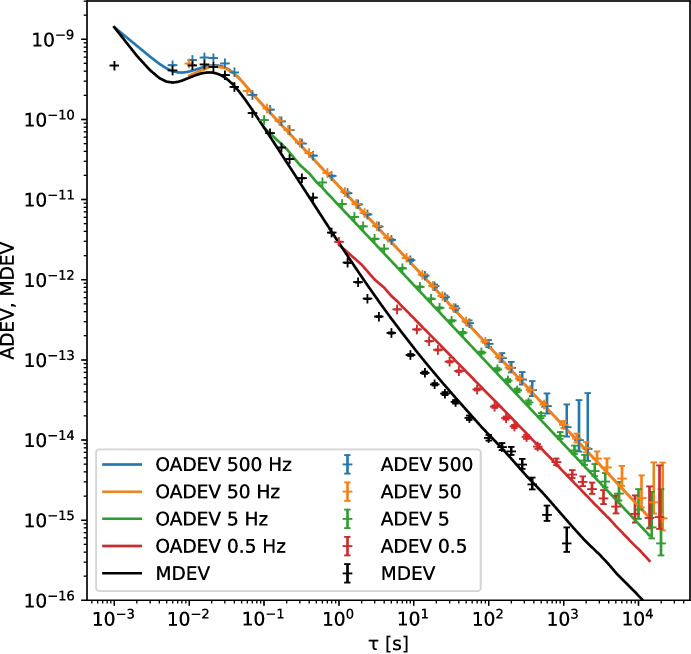
WR link without LJD. The lines are the ADEV and MDEV of the simulated noise, and the crosses with error-bars mark the measurements on the signal from the regular WR switches

**Fig. 6:**
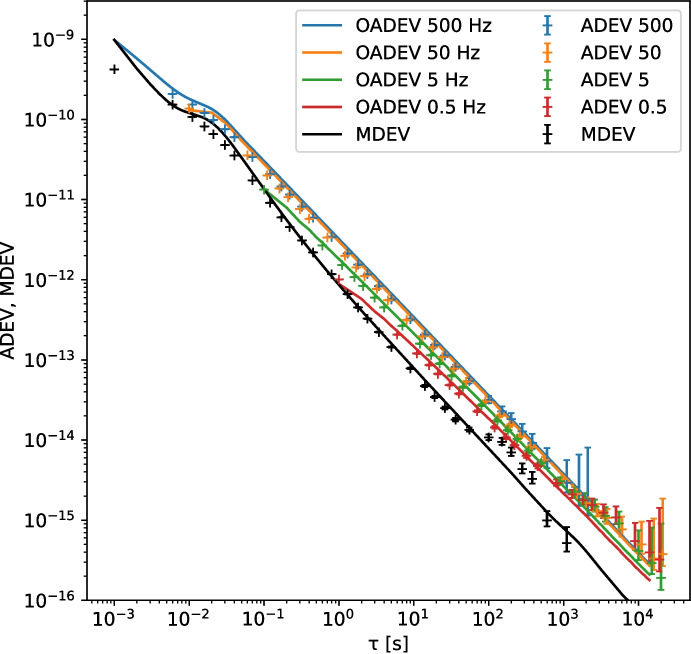
WR link with LJD. The lines are the ADEV and MDEV of the simulated noise, and the crosses with error-bars mark the measurements on the signal from the WR switches equipped with LJD

Figures 5 and 6 show the good correspondence between our measured and simulated values of the ADEV and MDEV for both types of WR switch.

Under the assumption that the phase noise contributions due to WPN and FPN are Gaussian and stationary, and therefore independent, their combined effect on the coherence loss can be described by

8 $$\begin{aligned} L_C = 1 - \sqrt{\langle C^2_\textrm{WPN} \rangle \cdot \langle C^2_\textrm{FPN} \rangle } \end{aligned}$$

**Fig. 7:**
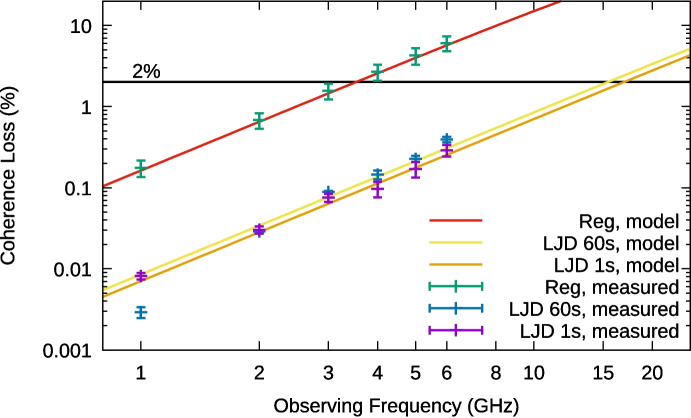
Direct coherence loss measurement results. The lines give the expected coherence loss for the regular WR link, and the coherence loss at 1 s and 60 s integration time for the LJD equipped links. The measurements with the SDR setup have error bars calculated by propagating the $1\sigma$ deviation of the $\langle C^2(T)\rangle$ measurements

Given the measured values for the noise parameters for both types of WR links, we can now predict the coherence loss as a function of observing frequency and integration time, as shown in Fig. 7 and summarized in Table 2 below.

**Table 2:** Highest observing frequency $\nu _0$ where $L_C < 2\%$

	WR	WR-LJD	
	1 s	1 s	60 s
$\nu _0 | L_C < 2\%$	3.5 GHz	17 GHz	15 GHz

### Direct measurement of coherence loss

In order to confirm these calculations of the coherence loss, we built a mock interferometer testbed using a signal generator and a software defined radio (SDR). It simulates a radio telescope receiving a signal, while its reference clock is supplied by the White Rabbit system.

Converting the 10 MHz reference clock to the observing frequency in the SDR can alter its phase noise characteristics, which needs to be taken into account. We use an Ettus Research / National Instruments B210 SDR in this experiment. From the schematic diagram of the B210 we know that the PLL loop-bandwidth of the local oscillator in the SDR when using an external reference is 4 kHz, and it will thus track any phase deviations with modulation frequencies below this value. As shown in Fig. 3, the phase noise at 4 kHz is down by more than 40 dB, so we can assume that this captures all the phase noise of the WR link.

**Fig. 8:**
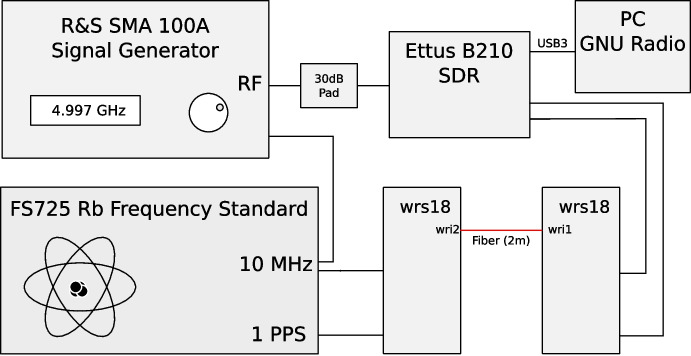
Testbed to measure the coherence loss. Connections are coaxial cables, unless marked otherwise

As depicted in Fig. 8, we use the signal generator to inject a sinusoidal signal at the observing frequency. The SDR, which receives its reference clock through WR, is tuned to 260 kHz below the observing frequency in order to avoid a possible ‘DC spike’ in the spectrum. The SDR down-converts the sinusoidal signal before digitizing it. Digital signal processing implemented in a GNU Radio flowchart then normalizes the amplitude of the input signal, shifts its frequency to DC, and determines the loss in amplitude after integrating the signal for an averaging time of 1 s or 60 s. Due to the normalization, the expected amplitude is 1. The measured reduction in amplitude is the coherence loss, which we plot as function of the observing frequency in Fig. 7, showing good agreement between the predicted and measured values for the coherence loss.

For the WR-LJD switches, the coherence loss is mostly due to FPN, and therefore also dependent on the integration time. These are measured at both 1 s and 60 s integration time.

**Fig. 9:**
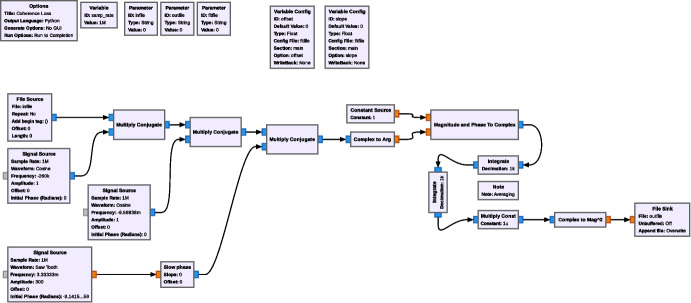
GNU Radio flow chart to measure the (mean squared) coherence $\langle C^2(T)\rangle$

The measurements require that the receiver chain is tuned to mHz accuracy relative to the generated sinusoid, as any phase rotation during an integration would add to the measured coherence loss. This turns out to be somewhat challenging, the major factor limiting the tuning accuracy is the limited frequency resolution of the ‘Signal Source’ GNU Radio block. In order to allow accurate phase stopping, we implemented a Python-based ‘Slow Phase’ block that can generate very low frequency offset frequencies, to 30 nHz accuracy. The GNU Radio flowchart for calculating the coherence from the measured data is depicted in Fig. 9.

We ran this testbed three times: with the regular WRS switches, with switches with the LJD modification, and with the WRS link replaced by coaxial cables, to determine the sensitivity limit of the setup. In our first measurements, the phase noise in the setup without WR was much higher than in any run using a WRS. It turned out that the +7 dBm output level of the 10 MHz from the FS725 is sufficient for the WRS, but not quite enough for the B210 SDR. This caused it to intermittently lose phase lock to its reference during the measurements. An additional reference amplifier (based around an 74HC06 IC) was built to remedy this. The phase noise of the B210 is listed as $1.5^\circ$ RMS at 6 GHz, which would correspond to about 0.03% coherence loss, much lower than the measured results at 6 GHz.

## Extending the range of White Rabbit

The White Rabbit design prescribes the use of 1000Base-BX10 optics, using wavelengths of 1310 nm and 1490 nm, which support distances of up to 10 km. Longer links can be achieved in several ways. WR links can be cascaded [27], optical transceivers with longer reach can be used, and the optical signal can be amplified. We will explore the latter two options in this section.

By replacing the prescribed 1000base-BX10 optics with longer reach, bidirectional optics available on the market, WR links can be extended up to 120 km. However, to achieve a longer reach, these must use wavelengths further away from the fiber chromatic dispersion minimum at 1310 nm on G.652.D fiber, and more towards its attenuation minimum at 1550 nm. Dispersion, i.e. the group velocity being dependent on the wavelength of the optical signal, causes slowly varying timing errors on a WR link due to the drift of the wavelength of the laser emitter in the Small Form-factor Pluggable (SFP) optical module. It can also, through the temperature dependence of the laser wavelength, deteriorate the frequency transfer stability.

For single mode optical fiber (G.652.D [14]), the dispersion at a wavelength of 1550 nm is typically $17\,\frac{\textrm{ps}}{\textrm{nm}\cdot \textrm{km}}$. As shown by us previously [7], the timing errors due to chromatic dispersion when using 80 km BiDi optics (1490/1550 nm) can be as high as 1.24 ns for a single 80 km link.

To mitigate this, we use wavelength stabilized optics intended for use in Dense Wavelength Division Multiplexing (DWDM) networks [28], which limit the drift of the laser wavelength to within 0.1 nm of their nominal wavelength, by employing o.a. active temperature stabilization for the laser.

The remaining wavelength variations limit the accuracy of the time transfer, with the resulting one-way timing variations $\delta t = D l \delta \lambda$ with the dispersion *D* in units of $\frac{\textrm{ps}}{\textrm{nm}\cdot {\textrm{km}}}$, $\delta \lambda$ the wavelength uncertainty in nm, and *l* the length of the fiber in km. The uncertainty of the time transfer due to one laser will be half this value, as the WR endpoint determines the one-way delay by (approximately) halving the measured round-trip time. However, the link uses a laser at each at each end with similar performance, and with uncorrelated errors the RMS timing variations on a WR link [16] would then be:

9 $$\begin{aligned} \delta t = \frac{D l}{\sqrt{2}}\delta \lambda \end{aligned}$$

On the same 80 km link, the use of DWDM stabilized optics thus reduces the time transfer variability due to SFP wavelength changes to 96 ps.

Although BiDi SFPs and DWDM SFPs are both CoTS equipment, there are no SFPs on the market which combine both features. Instead, we make use of DWDM stabilized SFPs, and use external Bragg based wavelength multiplexers to allow two counter-propagating wavelengths on the same fiber. As the DWDM SFP at each end of the link will be connected to such a multiplexer, any delay asymmetries cancel to below the ps level, and below our ability to measure them.

This system of using DWDM stabilized optics for WR links is in use (and has been widely tested) between the Dutch radio telescopes at Westerbork (WSRT) and Dwingeloo. It is also being implemented in the SKA radio telescope [12] which is currently under construction, and the LOFAR 2.0 upgrade [29].

### Chromatic dispersion calibration using conjugate wavelengths

In order to achieve the expected performance for the time and frequency transfer in a White Rabbit system, several calibration steps need to be undertaken as described in the White Rabbit calibration manual [10]. The fixed delays in the FPGA (field programmable gate array), PCB (printed circuit board) and SFP can be characterized in the lab, and are independent of the fiber in use. A WR system uses PTP to measure the total round trip time from timeTransmitter to timeReceiver and back. After subtracting all the known fixed delays, the remainder is the round trip time purely due to the optical path between the two devices. From this, the one-way delay from timeTransmitter to timeReceiver can be established, after taking the ratio of the propagation velocities for the two wavelengths in use into account. This difference in propagation velocity is due to the chromatic dispersion of the fiber, and depends on the characteristics of the fiber, and the two wavelengths in use.

The calibration parameter $\alpha$ relates the propagation velocities of the counter propagating signals in the same fiber, where the “out” direction corresponds to the optical signal going from the timeTransmitter to the timeReceiver, and the “return” signal will travel in the opposite direction on the same fiber.

10 $$\begin{aligned} \alpha = \frac{\delta _{\textrm{out}}}{\delta _{\textrm{return}}} -1 = \frac{v_g(\lambda _{\textrm{return}})}{v_g(\lambda _{\textrm{out}})} -1 \end{aligned}$$

with $\delta$ the delays on the fiber in each direction, and $v_g$ the group velocity at the wavelength in use.

Determining $\alpha$ on already installed fiber can be challenging. The fiber links in e.g. the SKA telescope which is currently under construction can each consist of a mix of ultra-low loss and bend insensitive fiber, which makes it difficult to predict their total dispersion in advance. The White Rabbit calibration manual describes the use of an additional fiber, a secondary electro-optical system to transmit a PPS signal over this fiber, and visiting both ends of the link with a time-interval counter or oscilloscope to determine the timing skew due to the dispersion. We simplify this by using two parallel White Rabbit links, with their wavelengths swapped, to determine the dispersion on the fiber. This eliminates the travel and use of additional equipment, and allows the measurement to be taken continuously while the link is operational. We require only one White Rabbit switch at each end of the link, two fibers, and two pairs of optical transceivers. The two fibers should have the same average dispersion, but do not need to have exactly the same length.

**Fig. 10:**
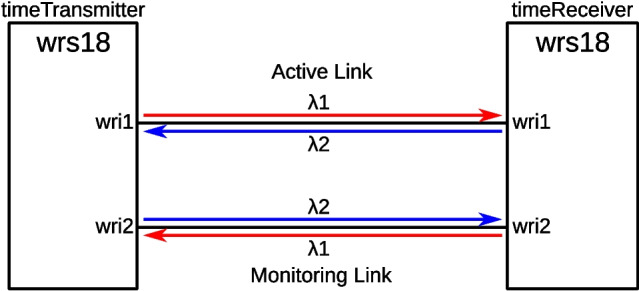
Dispersion calibration using two fiber links with swapped wavelengths

The regular firmware for the switches allows for only one port to be used as a timeReceiver, and steer its timebase. We introduce a new feature where a port can be designated as a ‘monitoring endpoint’, which participates in all the PTP message exchanges, but does not steer the clock on the switch. Our software then determines the clock steering that would be needed to align the monitoring link to match the timing from the active link. This offset $\textrm{Clock}^M_\textrm{offset}$ is equal to the difference in propagation time for the two forward wavelengths over the two parallel links. The White Rabbit system also measures the round trip times on the active ($d^A_{MM}$) an monitoring ($d^M_{MM}$) link, and from these three observables, we directly calculate and display the value of the dispersion calibration constant $\alpha$. Entering this value in the configuration of the link should reduce the corrected timing offset between the two channels to zero. Figure 10 shows the configuration of the dual link. The two dispersion calibration constants, as detailed in Appendix B, are then:

11 $$\begin{aligned} \alpha _{A}&= 4 \frac{\textrm{Clock}^M_\textrm{offset}}{-2\textrm{Clock}^M_\textrm{offset} + d^{A}_{MM} + d^{M}_{MM}}\end{aligned}$$

12 $$\begin{aligned} \alpha _{M}&= -4 \frac{\textrm{Clock}^M_\textrm{offset}}{2\textrm{Clock}^M_\textrm{offset} + d^{A}_{MM} + d^{M}_{MM}} \end{aligned}$$

Although $\alpha _A$ and $\alpha _M$ will return two different values as shown by the formula above, these two values will be related by (B16) when the two links are using the conjugated wavelength configuration. To the end-user, the value of $\alpha$ does not change when the wavelengths on a link are swapped, as the firmware reads out the transmit wavelength from the inserted SFP and applies the conversion only when needed.

On two identical fiber spools of 20 km length each, our system measures $\alpha _A = 4.357(4) \cdot 10^{-4}$ and $\alpha _M = 4.358(4) \cdot 10^{-4}$, after application of (B16). Using the published calibration procedure, we find $\alpha = 4.4(3) \cdot 10^{-4}$. The large improvement in the uncertainty is due to our method directly using the ps resolution and sub-ps accuracy [19] of the White Rabbit internal measurements, whereas the published method depends on the use of an external time interval counter, which in our case has a $1\sigma$ uncertainty of 85 ps.

Further confirmation of this method has been achieved by calibrating the dispersion of two looped-back fiber runs of 70 km each, using our simplified calibration method. In this test, we use DWDM stabilized optics operating at ITU channel C21 (1558 nm) and C23 (1560 nm) with a nominal reach of 80 km. External Bragg diplexers at each end of the link are used to put the counter-propagating wavelengths on a single strand of fiber. The setup is similar to that of Fig. 10, with the two switches located close to one another in the same rack. The fibers run together for 35 km underground, and then loop back to make two links of 70 km each. In this way, the time transfer performance can be measured by comparing the PPS outputs from the timeTransmitter and the timeReceiver switch using a time interval counter. At the same time the $\textrm{Clock}^M_\textrm{offset}$ is being read out from the timeReceiver.

This testbed uses buried G.655.D [13] (non-zero dispersion shifted) fiber, which has a dispersion of $4\pm 1 \mathrm{\frac{ps}{nm\cdot km}}$ at the ITU channels in use. As there are four SFPs, with four wavelengths for which we assume the errors are independent, this would result in an uncertainty of 5 ps on the monitoring link. We ignore the uncertainty in *D*, as it will be highly correlated between two identical fibers on identical paths. Likewise, the uncertainty in the length of the fibers is insignificant for this calculation, cancels out due to the calibration of the WR link, and any variability will again be correlated between the two fibers.

**Fig. 11:**
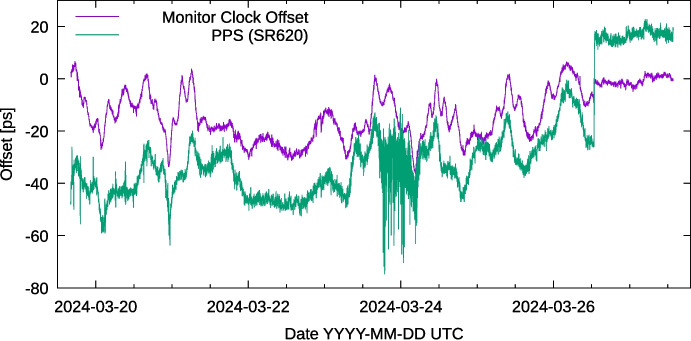
PPS offsets measured on two 70 km underground fibers. The active link with a time interval counter, and on the monitoring link using the clock offset on the WR switch itself. The values have been averaged over 100 seconds. At the discontinuity on 2024-03-26, the two long fiber links were replaced with two short fibers

Given these two rather close wavelengths in use, our software measures the dispersion correction $\alpha$ to be $1.74\cdot 10^{-6}$. After entering this value in the configuration of the WR switches, the system arrives at an initial timing offset of only 10 ps. On a timescale of hours, small non-linearities in the WR DDMTD timing measurement cause outliers of about 20 ps as can be seen in Fig. 11. Other contributions include polarization mode dispersion on the fiber link, and the measurement for the active link shows a drift due to the temperature sensitivity of the time interval counter used. At the start of the measurement (a few days after performing the dispersion calibration) this difference has become about 40 ps. At the discontinuity on 2024-03-26, the link was replaced by two short fibers of 2 m each, and two attenuators of 15 dB. The improvement on stability shows that the changes in PPS offset are not present when using a small length of fiber.

### Co-existence with production DWDM fiber networks

As part of the EU Horizons-2020 ASTERICS project, we set out to demonstrate that reference frequency distribution using White Rabbit does indeed offer sufficient stability for radio interferometry at cm wavelengths, over significant distances. We conducted Very Long Baseline Interferometry (VLBI) observations between two Dutch radio telescopes in Westerbork and Dwingeloo, and other radio telescopes in the EVN (European VLBI Network). Furthermore, our aim was to confirm that the White Rabbit optical signal can co-exist with other applications on the same fiber, within a modern production communications network employing DWDM technology, carrying high speed data traffic on many different wavelengths.

**Fig. 12:**
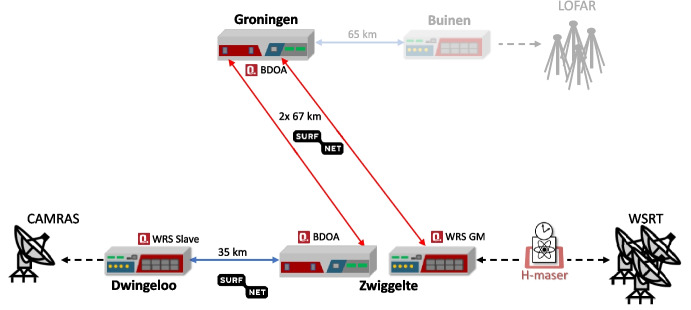
White Rabbit link between the WSRT and CAMRAS radio telescopes, taking a detour via Groningen. Also shown is the later extension to the LOFAR radio telescope, and the locations of the bi-directional optical amplifiers (BDOA) and WR grandmaster (GM) and slave switches

We built a White Rabbit link joining the Westerbork Synthesis Radio Telescope (WSRT) to the historic Dwingeloo radio telescope, both located in the Netherlands. The WSRT is a major radio instrument consisting of 14 dishes with a diameter of 25 m each, and is an active member of the EVN, participating regularly in VLBI observations. The Dwingeloo radio telescope is a single dish of 25 m diameter which is no longer in active scientific use, but is maintained and operated by the volunteer organization CAMRAS.^1^ The two telescopes are separated by 18 km geographically, and are connected by a 35 km dark fiber link consisting of multiple fibers. Both locations also host DWDM equipment for the Dutch research and educational provider SURF. This enabled us to build a much longer link by including a detour via their DWDM link to the city of Groningen, creating a link with a total distance of 169 km as depicted in Fig. 12.

**Fig. 13:**
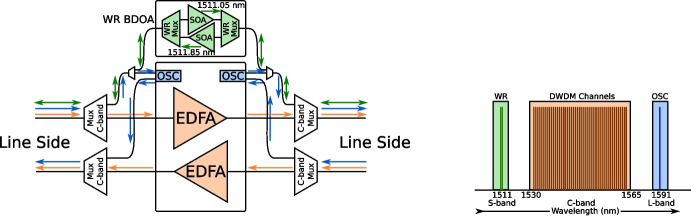
Left: A DWDM amplifier site with two uni-directional links and OSC, with a bi-directional WR link implemented on the eastwards direction of the link. Right: The wavelengths in use on this link

While telecommunication networks use separate fibers for the two directions of a network link, White Rabbit requires the use of the same fiber in both directions, making it incompatible with standard telecommunications equipment and practices. To sidestep this issue, we made use of the ability of the SURF optical networking equipment to separate out wavelengths that are outside of the C-band region, as depicted in Fig. 13. The C-band region of the spectrum spans a wavelength range of 1530 nm to 1565 nm. The signals in this wavelength range can be amplified by the use of erbium doped fiber amplifiers (EDFA), which is an important enabler for DWDM networks. For our long-haul White Rabbit link, we use wavelengths of 1511.05 nm and 1511.85 nm in the S band. To amplify these wavelengths while counter-propagating on the same fiber, we employ custom bi-directional silicon optical amplifiers (SOA) at the amplifier sites where the regular C-band traffic is amplified through a pair of one-directional EDFAs. Another out-of-band signal between the amplifier sites is the so-called optical supervisory channel (OSC), which is used to manage the equipment along the path. In this case, the OSC has been implemented at 1591 nm in the L band, to prevent interference with the White Rabbit time and frequency service on the fiber. As the installed optical equipment already separates out all the wavelengths outside of C band, we could add our equipment with only a brief interruption to the OSC, and without any interruption to the production data traffic in the C band.

## VLBI with White Rabbit

Using the long distance White Rabbit link described in the previous section, we embarked on a series of VLBI observations to characterize the performance of the time and frequency transport. Significant work was required to enable VLBI observations with the Dwingeloo radio telescope, including the installation of 275 m of single-mode fiber, and the design of a software based VLBI data formatter [6] to process the radio spectrum after digitizing it with a SDR. Initial observations were between the Dwingeloo and WSRT telescopes, and the Mark 2 at Jodrell Bank Observatory in the UK.

The default observing frequency at L-band for the EVN is 1650 MHz, but the feed system of the Dwingeloo telescope turned out to have insufficient sensitivity at this frequency, which caused additional phase noise in our measurements. We therefore performed additional observations at an observing frequency of 1330 MHz, where the Dwingeloo radio telescope has much higher sensitivity. These observations were performed together with the nearby WSRT radio telescope, and consisted of uninterrupted recordings of 1h30m each on the source 3C84. The length of the observations was needed to determine the ADEV performance with sufficient statistical accuracy out to an integration time of 15 minutes. Given the separation of only 18 km between these telescopes, and the correlation amplitude as function of UV distance for 3C84 [18], we treat the source as fully unresolved, expecting no structure based phase variations during our observations.

**Fig. 14:**
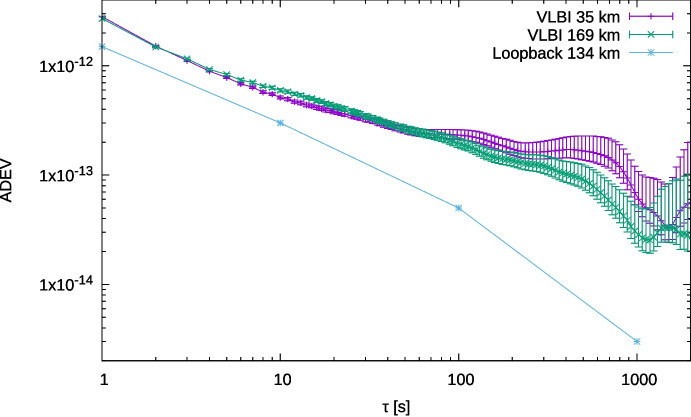
Allan deviation from the VLBI observations, compared to the WR link performance. ENBW = 0.5 Hz. The distances in the legend refer to the length of the WR link

For these observations, the data was correlated using the EVN SFXC [17] correlator at JIVE, and the resulting phase measurements were then converted into Allan deviations, as shown in Fig. 14. The phase noise performance of the WR link is lower than the measured ADEV using the VLBI method, indicating that, as intended, the WR link is not limiting the sensitivity of our VLBI observations. We used both a direct dark fiber link of 35 km between Westerbork and Dwingeloo, and the 169 km path on the SURF DWDM network of 169 km. These were performed at different times, yet their ADEV is essentially the same out to 100 s, showing that the link length in this case has no measurable effect on the phase noise performance.

## Conclusions

We have shown that White Rabbit is suitable for the distribution of a phase reference signal in radio interferometers, up to an observing frequency of 15 GHz when using low-jitter daughter-board equipped switches.

In order to determine this, we model the phase noise contribution of the White Rabbit link as a combination of white phase noise and flicker phase noise, and then calculate the coherence loss directly from the measured Allan deviation. Measuring the coherence loss in a testbed confirms these results.

We have introduced a simplified version for the calibration of the dispersion of a fiber, which brings more convenience and accuracy, and have demonstrated a method which allows the WR traffic to be carried by production telecommunication networks equipped with DWDM technology.

Finally, we have illustrated the usefulness of the above results by performing VLBI observations with the reference clock signal co-existing on the same fiber with high-speed data traffic.

## Data Availability

No datasets were generated or analysed during the current study.

## Appendix A: Derivation of coherence due to flicker phase noise

To determine the coherence loss due to flicker phase noise, we follow the method described in [26]. Coherence can be expressed as a function of the true variance $I^2(\tau )$:

A1 $$\begin{aligned} \langle C^2(T)\rangle = \frac{2}{T}\int _0^T \left( 1-\frac{\tau }{T} \right) e^{-\pi ^2 \nu _0^2 \tau ^2 2I^2(\tau )}\textrm{d}\tau \end{aligned}$$

The true variance $I^2(\tau )$ can be expressed as an infinite sum of Allan variances:

A2 $$2I^2(\tau )= \sigma ^2_y(\tau ) + \sigma ^2_y(2\tau ) + \sigma ^2_y(4\tau ) + \cdots = \sum _{n=0}^\infty \sigma _y^2(2^n\tau )$$

For a simple power law behavior of the AVAR, the above is a geometric series and will converge as long as the slope of the AVAR/ADEV plot is negative.

The Allan Variance for flicker phase noise [4]:

A3 $$\begin{aligned} \sigma _y^2(\tau ) = \frac{3\gamma - \ln 2 + 3 \ln (2\pi f_h\tau )}{4\pi ^2}\frac{h_1}{\tau ^2} \end{aligned}$$

with $\gamma$ = Euler-Mascheroni constant, $0.57721\cdots$

A4 $$\begin{aligned} 2I^2(\tau ) = \sum _{n=0}^\infty \frac{3\gamma - \ln 2 + 3 \ln (2\pi f_h 2^n \tau )}{4\pi ^2}\frac{h_1}{(2^n\tau )^2} \end{aligned}$$

Splitting off a factor of $3 \ln 2^n$ from the second logarithmic term in the above formula leads to a convenient cancellation of the $\ln 2$ terms. We make use of these infinite sums:

A5 $$\begin{aligned} \sum _{n=0}^\infty \frac{1}{4^n}= & \sum _{n=0}^\infty \frac{3n}{4^n} = \frac{4}{3}\end{aligned}$$

A6 $$\begin{aligned} 2I^2(\tau )= & \frac{3 h_1}{4 \pi ^2 \tau ^2}\sum _{n=0}^\infty \frac{\gamma + (n - \frac{1}{3})\ln 2 + \ln (2\pi f_h\tau )}{4^n} \nonumber \\= & (\gamma + \ln (2\pi f_h\tau ))\frac{h_1}{\pi ^2\tau ^2} = \frac{h_1 \ln (2\pi e^\gamma f_h\tau )}{\pi ^2\tau ^2} \end{aligned}$$

Substituting $2I^2(\tau )$ in the expression for the mean-squared coherence:

A7 $$\begin{aligned} \langle C^2(T)\rangle= & \frac{2}{T}\int _0^T \left( 1-\frac{\tau }{T} \right) e^{-h_1\nu _0^2\ln (2\pi e^\gamma f_h\tau )}\textrm{d}\tau \nonumber \\= & \frac{2(2\pi e^\gamma f_h)^{-h_1\nu _0^2}}{T}\int _0^T \left( 1-\frac{\tau }{T} \right) \tau ^{-h_1\nu _0^2}\textrm{d}\tau \end{aligned}$$

The integral converges for $h_1\nu _0^2 < 1$. At this upper frequency edge, the coherence loss will already be more than 90%.

A8 $$\begin{aligned} \langle C^2(T)\rangle = \frac{2(2\pi e^\gamma f_hT)^{-h_1\nu _0^2}}{(1 - h_1\nu _0^2)(2 - h_1\nu _0^2)} \end{aligned}$$

## Appendix B: $\alpha$ for a conjugate wavelength WR link

We begin by deriving a relation between the ideal master to slave clock offset $\mathrm {Clock^{ideal}_{offest}}$ and the one measured by White Rabbit $\mathrm {{Clock}_{offset}}$. Following the White Rabbit PTP formulae, the clock offset ($\mathrm {{Clock}_{offset}}$) between the WR-master and WR-slave can be calculated as:

A9 $$\begin{aligned} \textrm{Clock}_\textrm{offset} = t_1 - t_2 + d_{MS} \end{aligned}$$

Here $t_1$ and $t_2$ are the WR-PTP timestamps measured at master and slave respectively, and $d_{MS}$ is the master to slave propagation delay on the fiber. Note that $d_{MS}$ is not directly measured, but inferred from the round-trip delay based on the transmission and reception delays. If the fiber asymmetry has been correctly calibrated, the ideal master to slave propagation delay ($d^\textrm{ideal}_{MS}$) is known and equation (B9) will yield the ideal clock offset $\textrm{Clock}_\textrm{offset}^\textrm{ideal}$ as:

A10 $$\begin{aligned} \textrm{Clock}_\textrm{offset}^\textrm{ideal} = t_1 - t_2 + d_{MS}^\textrm{ideal} \end{aligned}$$

However, if the fiber asymmetry is not known and set to zero, the master to slave clock offset is calculated using the round trip delay, $d_{MM} /2$, and the previous equation becomes:

A11 $$\begin{aligned} \textrm{Clock}^\mathrm {calc.}_\textrm{offset} = t_1 - t_2 + d_{MM} /2 \end{aligned}$$

Subtracting the previous two equations we obtain a relation between the ideal and measured clock offset:

A12 $$\begin{aligned} \textrm{Clock}^\textrm{ideal}_\textrm{offset} - \textrm{Clock}^\mathrm {calc.}_\textrm{offset} = d^\textrm{ideal}_{MS} - d_{MM} / 2 \end{aligned}$$

The previous equations are valid for both links, active and monitor. So we can use it to relate the clock offset measured at the active link, with that of the monitor link as:

A13 $$\begin{aligned} \textrm{Clock}^\textrm{ideal}_\textrm{offset}= & d^{A,\textrm{ideal}}_{MS} - d^A_{MM}/2 + \textrm{Clock}^{A,\mathrm {calc.}}_\textrm{offset}\nonumber \\= & d^{M,\textrm{ideal}}_{MS} -d^M_{MM}/2 + \textrm{Clock}^{M,\mathrm {calc.}}_\textrm{offset} \end{aligned}$$

Here we have introduced the additional superscripts M and A, to denote the clock offsets and fiber delays calculated using the monitor and the active links respectively. Now typically, White Rabbit steers the local time such that the clock offset of the active link becomes 0. Therefore the previous equation can be written solely in terms of the clock offset measured in the monitor link as:

A14 $$\begin{aligned} \textrm{Clock}_\textrm{offset}^{M,\mathrm {calc.}} = d_{MS}^{A,\textrm{ideal}} - d_{MS}^{M,\textrm{ideal}} - (d^A_{MM} - d^M_{MM})/2 \end{aligned}$$

From the definition of the fiber asymmetry, $\alpha = (d_{MS} - d_{SM})/d_{MS}$, we can easily obtain the following relations:

A15 $$\begin{aligned} d_{MS}^\textrm{ideal}= & \frac{\alpha +1}{\alpha +2}d_{MM}\end{aligned}$$

A16 $$\begin{aligned} \alpha _A= & -\frac{\alpha _M}{\alpha _M+1} \end{aligned}$$

The three previous equations give rise to a system of four independent equations with four unknowns. After solving for $\alpha _M$ and $\alpha _A$ we obtain these two equations:

A17 $$\begin{aligned} \alpha _{A}&= 4 \frac{\textrm{Clock}^M_\textrm{offset}}{-2\textrm{Clock}^M_\textrm{offset} + d^{A}_{MM} + d^{M}_{MM}}\end{aligned}$$

A18 $$\begin{aligned} \alpha _{M}&= -4 \frac{\textrm{Clock}^M_\textrm{offset}}{2\textrm{Clock}^M_\textrm{offset} + d^{A}_{MM} + d^{M}_{MM}} \end{aligned}$$

## Footnotes

1 https://www.camras.nl/en/
